# Supplementary material for: Reconstructing donor genotypes from scRNA-seq for downstream eQTL and HLA–TCR analysis
Source: bioRxiv. 2026 Oct 5:2026.09.30.755703. Preprint. [Version 1] doi: 10.64898/2026.09.30.755703 (PMC13691981; doi:10.64898/2026.09.30.755703)
Supplement: Supplementary file 1 [file media-2.pdf]

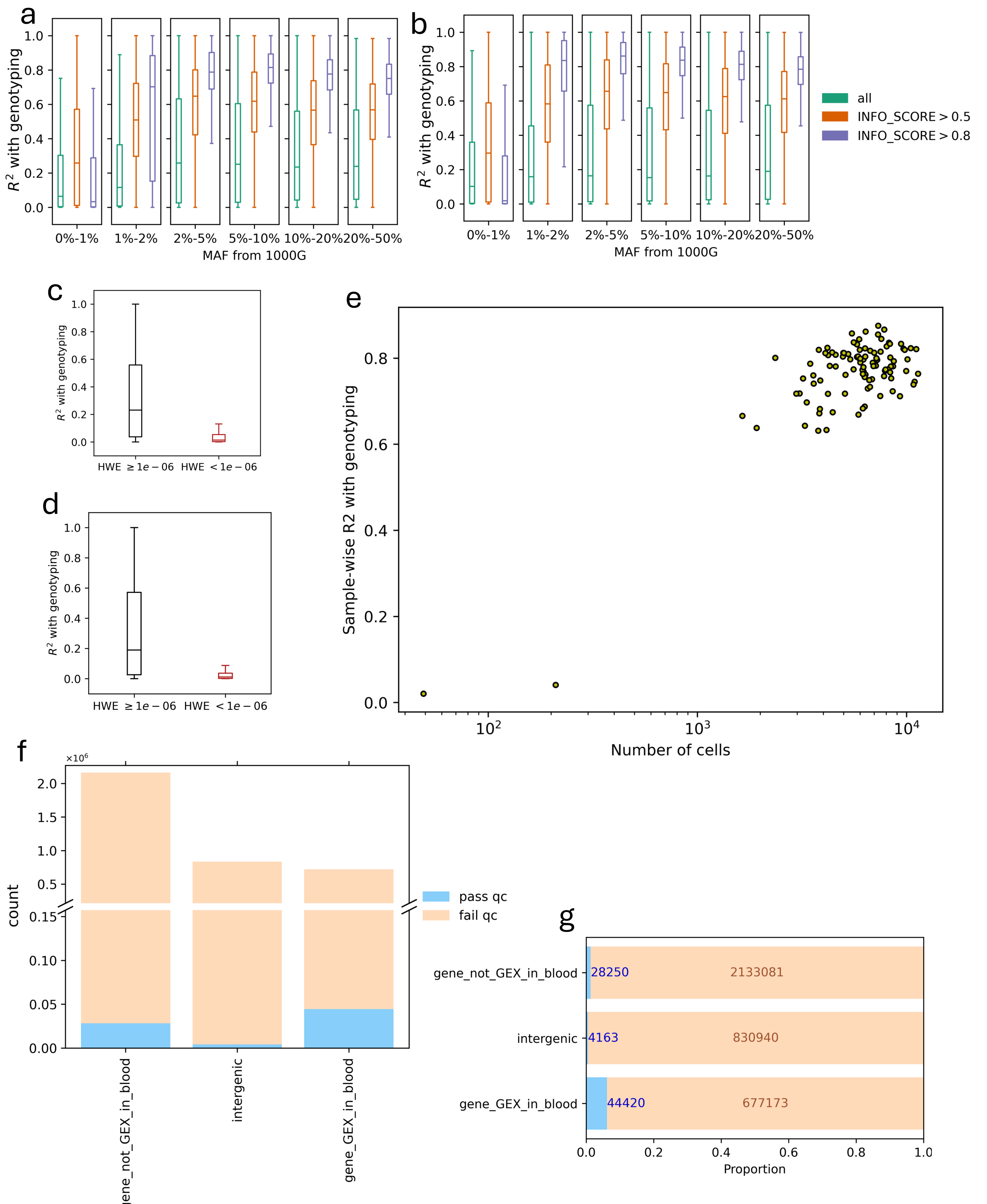

Fig S1. Genoype dosage concordance across minor allele-frequency (MAF) bins calculated using 1000 Genomes Project and INFO_SCORE filtering thresholds for GSA variants (**a**) and TOPMed-imputed variants (**b**). Genotype concordance above and below HWE threshold for GSA variants (**c**) and TOPMed-imputed variants (**d**). Sample-wise genotype concordance across number of cells (**e**). Number (**f**) and proportion (**g**) of variants annotated to genes that are expressed or not, or otherwise, that pass or fail quality control.

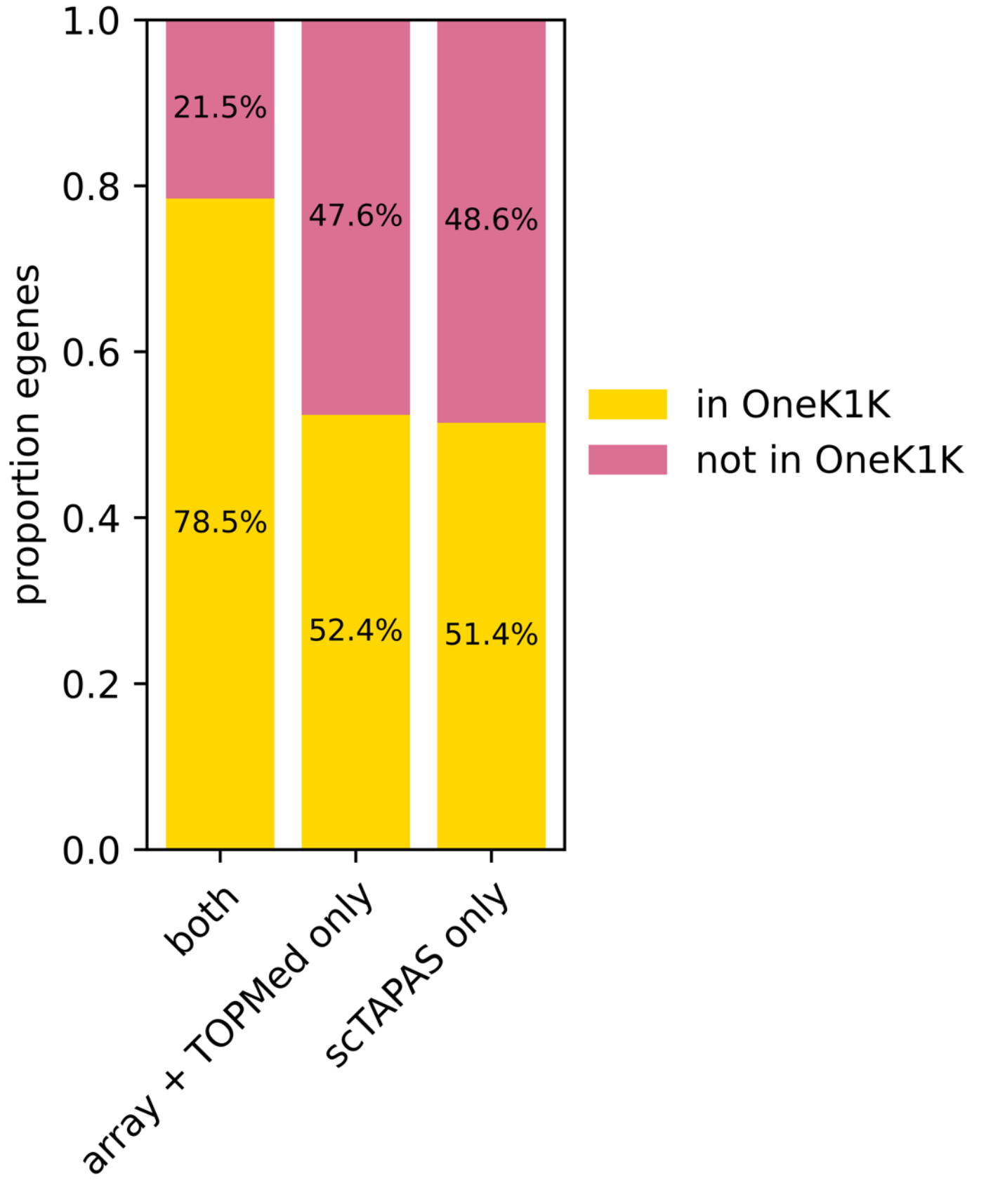

Fig S2. Proportion of eGenes overlapping with OneK1K for at least one matching cell type

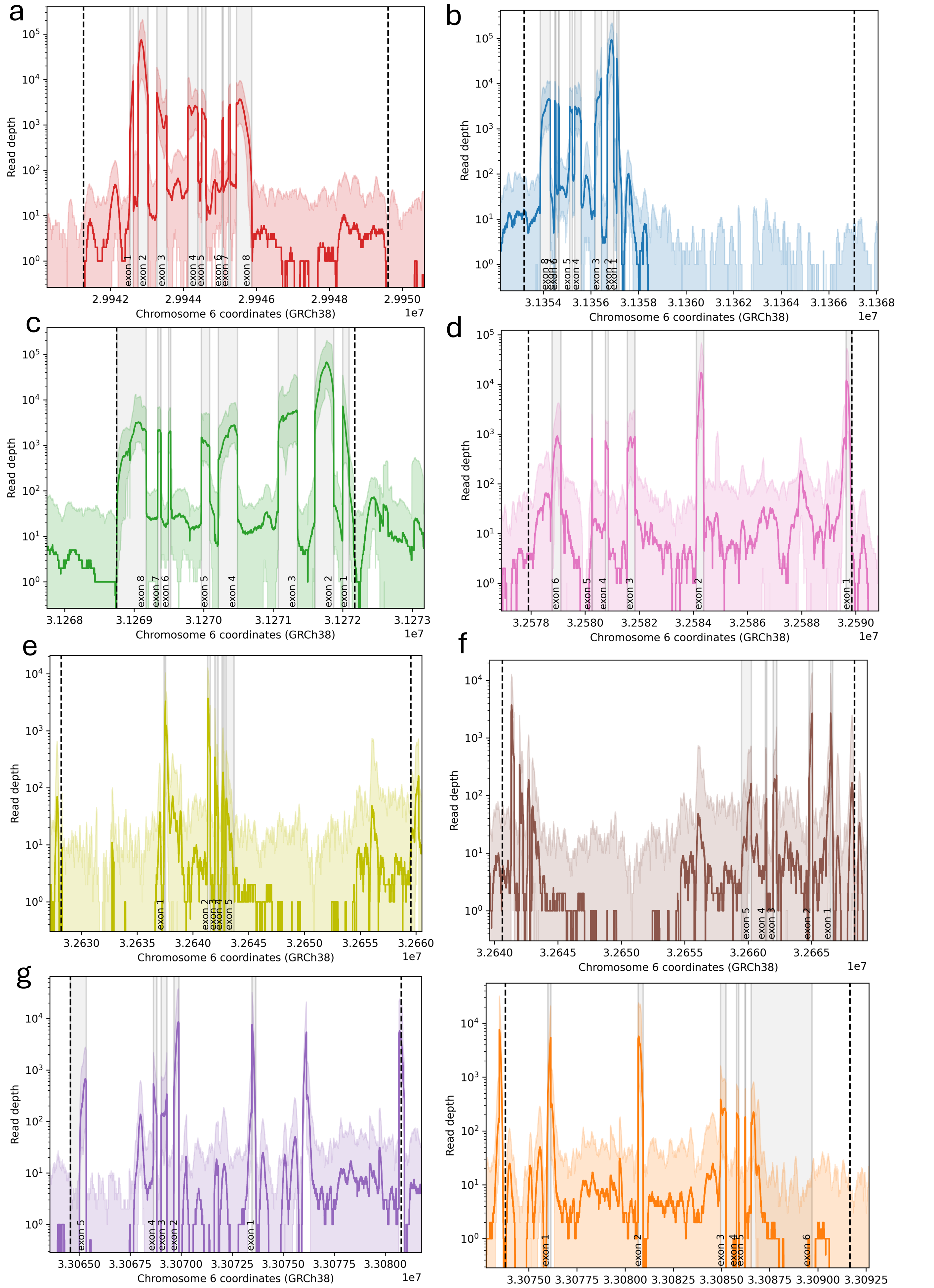

Fig S3. Read depth over classical HLA gene regions +-1kbp: HLA-A (**a**), HLA-B (**b**), HLA-C (**c**), HLA-DRB1 (**d**), HLA-DQA1 (**e**), HLA-DQB1 (**f**), HLA-DPA1 (**g**), HLA-DPB1 (**h**). Median shown in bold, minimum and maximum filled above and below.

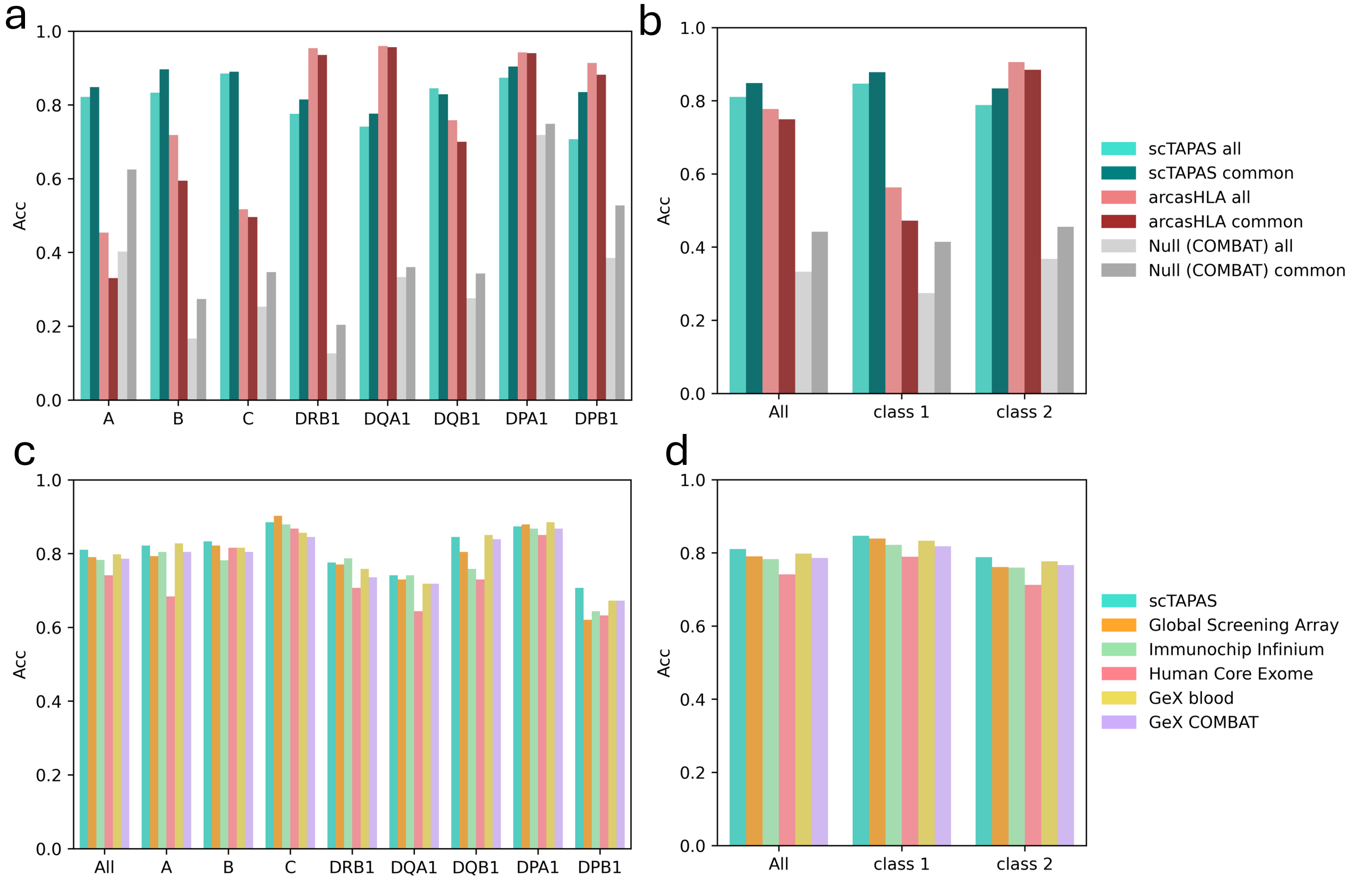

Fig S4. HLA gene-wise (**a**) and summarised gene-wise (**b**) accuracy for scTAPAS, arcasHLA and null model for all and common HLA alleles at one-field. HLA gene-wise (**c**) and summarised gene-wise (**d**) accuracy for scTAPAS and variations of scTAPAS where subsets of variants are extracted prior to HLA imputation.
